# LTA1 is a safe, intranasal enterotoxin-based adjuvant that improves vaccine protection against influenza in young, old and B-cell-depleted (μMT) mice

**DOI:** 10.1038/s41598-019-51356-w

**Published:** 2019-10-22

**Authors:** E. Valli, A. J. Harriett, M. K. Nowakowska, R. L. Baudier, W. B. Provosty, Z. McSween, L. B. Lawson, Y. Nakanishi, E. B. Norton

**Affiliations:** 1 Department of Microbiology & Immunology, Tulane University School of Medicine, New Orleans, USA; 2 Department of Pathology & Laboratory Medicine, Tulane University School of Medicine, New Orleans, USA

**Keywords:** Mucosal immunology, Adjuvants

## Abstract

Enterotoxin-based adjuvants including cholera toxin and heat-labile toxin (LT) are powerful manipulators of mucosal immunity; however, past clinical trials identified unacceptable neurological toxicity when LT or mutant AB_5_ adjuvant proteins were added to intranasal vaccines. Here, we examined the isolated enzymatic A1 domain of LT (LTA1) for intranasal safety and efficacy in combination with influenza (flu) vaccination. LTA1-treated mice exhibited no neurotoxicity, as measured by olfactory system testing and H&E staining of nasal tissue in contrast with cholera toxin. In vaccination studies, intranasal LTA1 enhanced immune responses to inactivated virus antigen and subsequent protection against H1N1 flu challenge in mice (8-week or 24-months). In addition, lung H1N1 viral titers post-challenge correlated to serum antibody responses; however, enhanced protection was also observed in μMT mice lacking B-cells while activation and recruitment of CD4 T-cells into the lung was apparent. Thus, we report that LTA1 protein is a novel, safe and effective enterotoxin adjuvant that improves protection of an intranasal flu vaccination by a mechanism that does not appear to require B-cells.

## Introduction

Vaccination is the most cost-effective method to prevent infectious diseases, yet 2.5 million deaths occur from vaccine-preventable illnesses annually^1^. Contributing to this problem are poor responsiveness to vaccines at the extremes of life and deficient protection at mucosal surfaces, as highlighted by recent influenza, pertussis, and polio studies^2–6^. In addition, vaccines are still needed for major intractable infections like HIV and tuberculosis^7^. Intranasal (IN) vaccination is an attractive delivery route due to the ease of administration (including possibility of self-administration), lack of injection-related infections, low antigen doses, and induction of robust systemic and mucosal immunity^8–10^. Adjuvants, such as aluminum salts, are useful additives to promote immunity to injected antigens. However, safe and effective IN adjuvants are lacking.

Perhaps the best-studied mucosal adjuvants are the enterotoxin proteins including heat-labile toxin *E*. *coli* (LT), cholera toxin (CT), and their derivatives^8,11–13^. To circumvent oral toxicity, LT was investigated for clinical use by nasal delivery. During 2000–2001, an LT-adjuvanted IN influenza vaccine was commercially available in Switzerland (Nasalflu, Berna Biotech). While efficacious, it was pulled from the market after a few months for safety issues; a subsequent investigation revealed a 19-fold higher risk of Bell’s palsy or facial paralysis in vaccine recipients^14^. Similar findings were observed with a detoxified mutant protein (LT-S63K)^14,15^. More recently, another mutant adjuvant, dmLT (LT-R192G/L211A), has exhibited success in Phase 1 and 2 clinical trials by non-IN mucosal and parenteral delivery routes^11^. Thus, LT adjuvants are potent mucosal adjuvants, but there is no candidate being considered for IN use.

LT and CT have an AB_5_ structure with an enzymatic A-subunit that is non-covalently associated with a pentameric B-subunit. The B-subunit is responsible for receptor binding and cellular entry by binding to cell surface gangliosides, particularly GM1^16^. The A-subunit can be proteolytically cleaved, creating an A1 domain that binds to cytosolic ADP-ribosylation factors and ADP-ribosylates the heterotrimeric G protein subunit Gsα. This leads to irreversible adenylate cyclase activation, cAMP accumulation, and protein kinase A (PKA) activation, inducing target protein phosphorylation^11^. In intestinal epithelial cells, this signaling cascade results in dysregulation of cell membrane ion channels and ultimately intestinal secretion. However, in immune cells stimulated during vaccination (e.g., LT or dmLT admixed with antigen), this leads to promotion of antigen-specific immune responses, including antibodies (IgG, IgA) and multipotent CD4 T-helper (Th)1/Th17/Th2 responses in both systemic and mucosal tissue compartments^11^. These adjuvant properties have been linked to stimulation of antigen-presenting cells (APCs)^17,18^ and inflammasome signaling^19–21^.

The mechanisms underlying development of Bell’s palsy by enterotoxin-associated adjuvants are thought to involve both GM1-binding by the B-subunit and inflammation associated with the A-subunit within olfactory neuroepithelium. Antigen trafficking into the olfactory neuroepithelium can be blocked with pre-incubation of CT or LT with recombinant GM1^22^. In addition, alterations in histopathology of the neuroepithelium and reduced neuronal firing and olfactory system function is observed with CT but not enzymatically inactive CT or purified B-subunit^23^.

Therefore, one strategy to create a safe IN LT adjuvant is to remove the B-subunit. To that end, we recently began investigating the A-subunit and the A1 domain of LT (LTA1) for use as adjuvants. Both LTA1 and A-subunit proteins have ADP-ribosyltransferase activity, but, unlike LT, no GM1 binding by ELISA or gastrointestinal toxicity by patent mouse assay^24^. Moreover, both proteins improved IN vaccine responses to co-delivered tetanus toxoid or ovalbumin in mice in contrast to non-enzymatically active LT B-subunit. By comparing combinations of LT proteins and subunits, we also observed that quality of the immune response (e.g., IgG1/IgG2 balance, mucosal IgA, and Th17 induction) was dependent upon the presence of an A-subunit. Additionally, while anti-A antibodies can neutralize LT toxicity, LTA1 is not a good antigen (in comparison with LTA and LT) and does not induce robust autoantibodies^25^. These studies highlighted two properties that make LTA1 unique among LT- and CT-derived adjuvants: low antigenicity and lack of a B-subunit or ganglioside binding. In the current investigation, we expanded upon these early studies and tested the hypothesis that LTA1 is a safe, effective adjuvant for IN vaccination to enhance protection from disease using a flu model.

## Results

### LTA1 does not cause neurological toxicity as observed with nasal administration of AB_5_ enterotoxins

**Figure 1:**
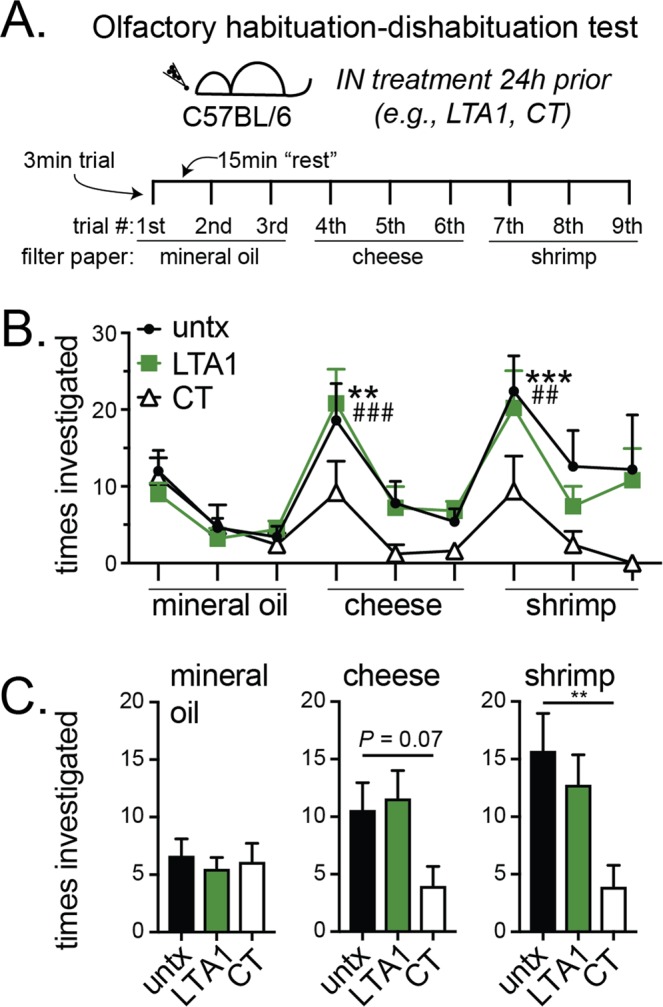
LTA1 does not cause nasal toxicity – reduced olfactory system function – observed with AB_5_ enterotoxins. (**A**) Schematic of habituation-dishabituation test: individually housed mice were untreated (untx) or IN treated with 30 μg LTA1 or 5–10 µg CT 24 h before behavioral testing with 3 min trials spaced 15 min apart. During each trial, mice were exposed to filter paper laced with mineral oil, cheese odor or shrimp odor as indicated. (**B**) Mean + SEM times investigating filter paper during each trial from 3 experiments totaling 5 mice/group. Significance is indicated (*untx, ^#^LTA1, **^,##^*P* ≤ 0.01, ***^,###^*P* ≤ 0.001) for trials with increased investigations compared to the habituation control (3^rd^ trial) using two-way repeated measures ANOVA with Dunnett’s post-test. No CT trials were significantly different than habituation control. (**C**) Mean + SEM times investigating filter paper compiled by odor for all three trials. Significance is indicated (**P ≤ 0.01) using ANOVA with Dunnett’s post-test compared to untreated.

Previous nasal vaccines with LT adjuvants were found to be unacceptable due to neurological toxicity that was not apparent in pre-clinical studies^14^. A newer methodology to evaluate IN toxicity of enterotoxins is olfactory behavioral testing which identifies effects on cranial nerve 1^23^. To verify our hypothesis that the lack of a B-subunit and GM1-binding precludes development of neurological toxicity after LTA1 IN administration, we performed a habituation-dishabituation test for olfactory system function (Fig. 1A). In this test, mice are first habituated to filter paper placement in their cage (containing mineral oil) then evaluated for subsequent investigation of new filter papers containing novel odors. In pilot analyses, we determined that 5–10 μg of CT IN was optimal for detection of olfactory system decline 24 h later (as higher doses limited mouse movement due to respiratory distress and lower doses were comparable to naïve mice, see Supplementary Fig. 1). We then treated mice with CT or 30 μg LTA1 IN and 24 h later performed the olfactory habituation-dishabituation test. Unlike CT-treated mice, LTA1-treated and untreated mice investigated cheese or shrimp odors significantly more times than their habituation control (i.e., in comparison with their third trial with mineral oil; Supplementary Video 1 and Fig. 1B). Compilation of all times investigating specific odors (from three trials) also indicated non-significant difference from LTA1-treated mice and untreated, whereas significantly lower investigations were observed with CT-treated mice in cheese and shrimp trials (Fig. 1C, respectively *P* = 0.07 or *P* < 0.01).

**Figure 2:**
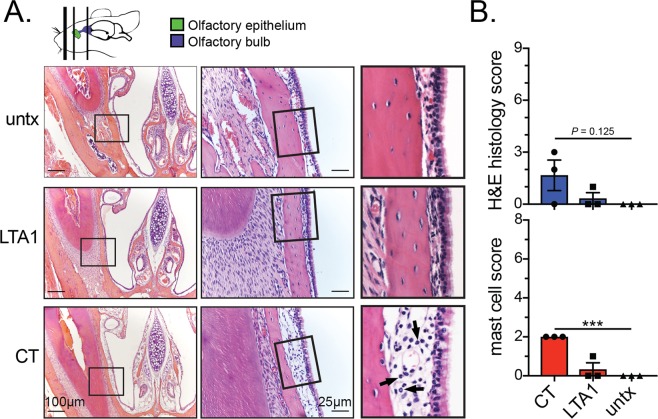
LTA1 does not cause mild inflammation or mast cell infiltration in nasal passages observed with AB_5_ enterotoxin CT. (**A**) Representative H&E-stained images of the nasal cavity of mice treated as indicated. Sections from location immediately posterior to the incisors, shown as left-most line on mouse diagram, olfactory epithelium (OE), and olfactory bulb (OB). Arrows indicate neutrophils and mild, acute inflammation beneath the epithelium within the nasal mucosa. (**B**) Composite histology score for induration and neutrophil infiltration in nasal tissue. (**C**) Composite score for mast cell numbers within nasal tissue.

Although major histological changes in nasal tissue were not previously been reported with LT^26^, we also further examined mice post-behavioral testing for histopathologic changes in three H&E- or toluidine blue-stained transverse sections through the nasal cavity according to established guidelines^27^ and depicted in Fig. 2A (top). CT-treated mice exhibited neutrophil infiltration (arrows) and mild, acute inflammation in subepithelial tissue of nasal passages immediately posterior to the incisors and at the level of the incisive papilla but not by the second molar tooth and also a significantly higher level of mast cells (Fig. 2A,B and Supplementary Fig. 2). No significant inflammatory changes were detected in nasal passages of LTA1-treated or untreated mice. Thus, LTA1 exhibits improved neurological safety compared with an AB_5_ enterotoxin after IN delivery.

### Immune responses to IN influenza immunization are enhanced with LTA1 adjuvant and exhibit dose-dependent responses for antibody generation

**Figure 3:**
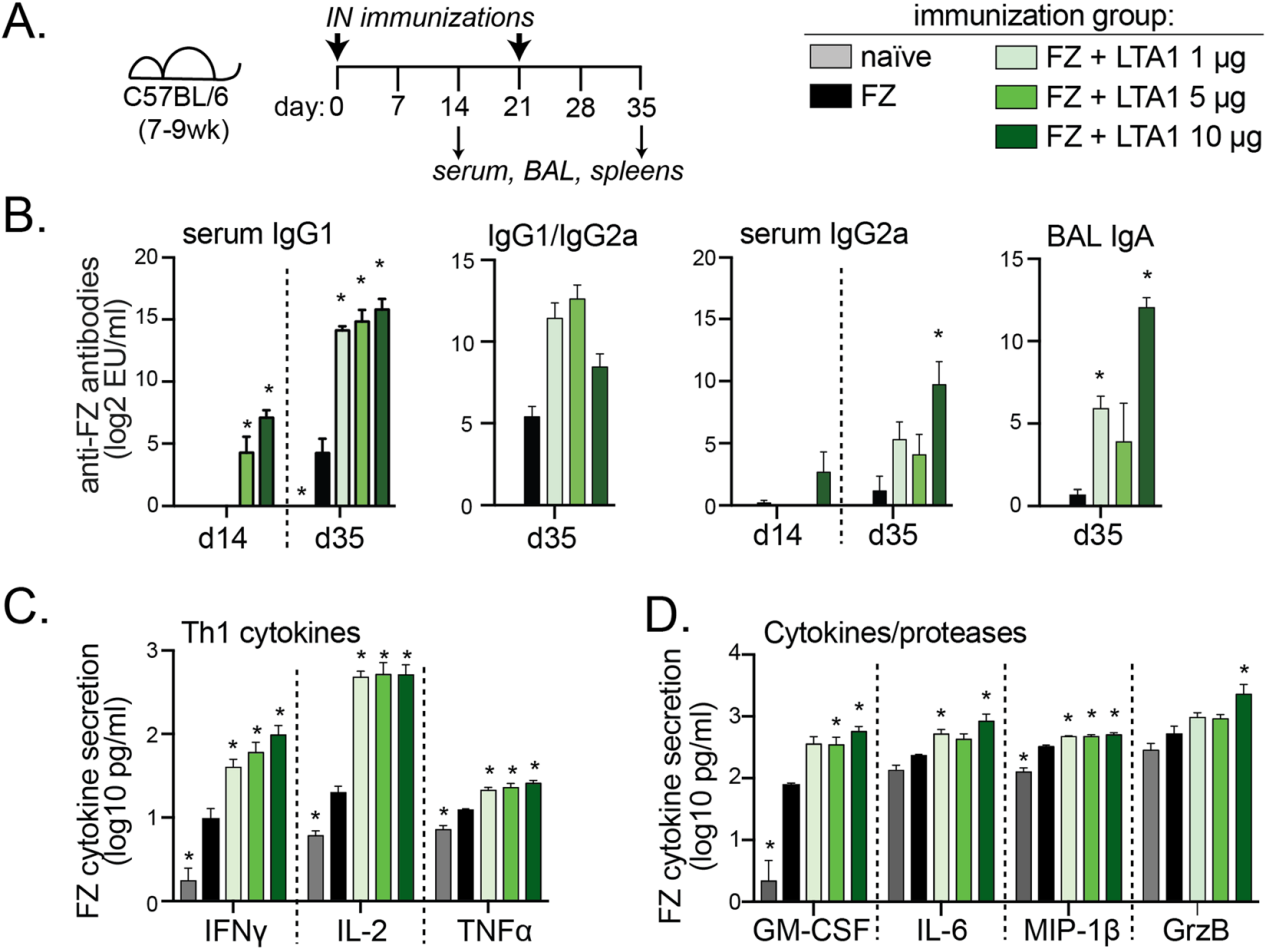
Immune responses to IN flu immunization are enhanced with LTA1 adjuvant. (**A**) Schematic of animal immunizations. Groups of C57Bl/6 mice (n = 5) were immunized intranasally with 3.6 μg HA-content Fluzone-HD (FZ) alone or with LTA1 doses as indicated. (**B**) Serum anti-FZ IgG1, IgG2a, IgG1/IgG2a ratio and BAL IgA ELISA results. The latter graph includes one animal in FZ + LTA1 10 μg that maxed out the assay and is approximated as highest value detectable. (**C**) Th1 cytokines and (**D**) Other cytokines/proteases (GrzB = granzyme B) from 72 h culture supernatants of FZ stimulated splenocytes from mice post-boost (day 35) (excluding outlier from FZ + LTA1 10 μg group). Significance (*) is indicated for *P* ≤ 0.05 for all groups compared to FZ by one-way ANOVA with Dunnett’s post-test.

To investigate whether LTA1 can boost immunity to an IN vaccine, we used the inactivated antigen from the injectable split-virus commercial flu vaccine Fluzone® (FZ). FZ formulations at 3.6 μg total hemagglutinin (HA) content were admixed with 1, 5 or 10 μg LTA1 and delivered by prime/boost IN immunization to mice (days 0, 21) with sample analyses performed two weeks after either immunization (day 14 or 35) (Fig. 3A). For all analyses, two doses yielded higher levels of antibodies and recall cytokine responses than one and the addition of LTA1 adjuvant increased detectable FZ-specific responses (Fig. 3 and Supplementary Fig. 3). Significant induction of serum IgG1 in the FZ group was observed compared to naïve animals on day 35, but only FZ + LTA1 groups developed antigen-specific serum IgG2a or bronchoalveolar lavage fluid (BAL) IgA. In general, development of antibody responses was adjuvant dose-dependent and maximal levels were observed with the highest dose of adjuvant (dark green bars, FZ + LTA1 10 μg, Fig. 3B).

We also evaluated memory cytokines responses to recall antigen using *ex vivo* cultured splenocytes (Fig. 3C,D). Robust cytokine secretion was observed in the FZ group compared to the naïve group including Th1 cytokines (INFγ, IL-2, TNFα) and other cytokines or proteases linked to protection from flu (GM-CSF^28^, MIP-1β). Inclusion of LTA1 augmented these responses and also promoted generation secreted factors IL-6 and Granzyme B (a serine protease that contributes to apoptosis of infected cells)^29,30^. However, with a few exceptions (e.g., Granzyme B, GM-CSF), the level of cytokine secretion was not adjuvant dose-dependent. Thus, pursuant to these results, we chose the 10 μg LTA1 dose for subsequent animal studies.

### LTA1 adjuvant enhances protection from H1N1 flu challenge in FZ-vaccinated mice

**Figure 4:**
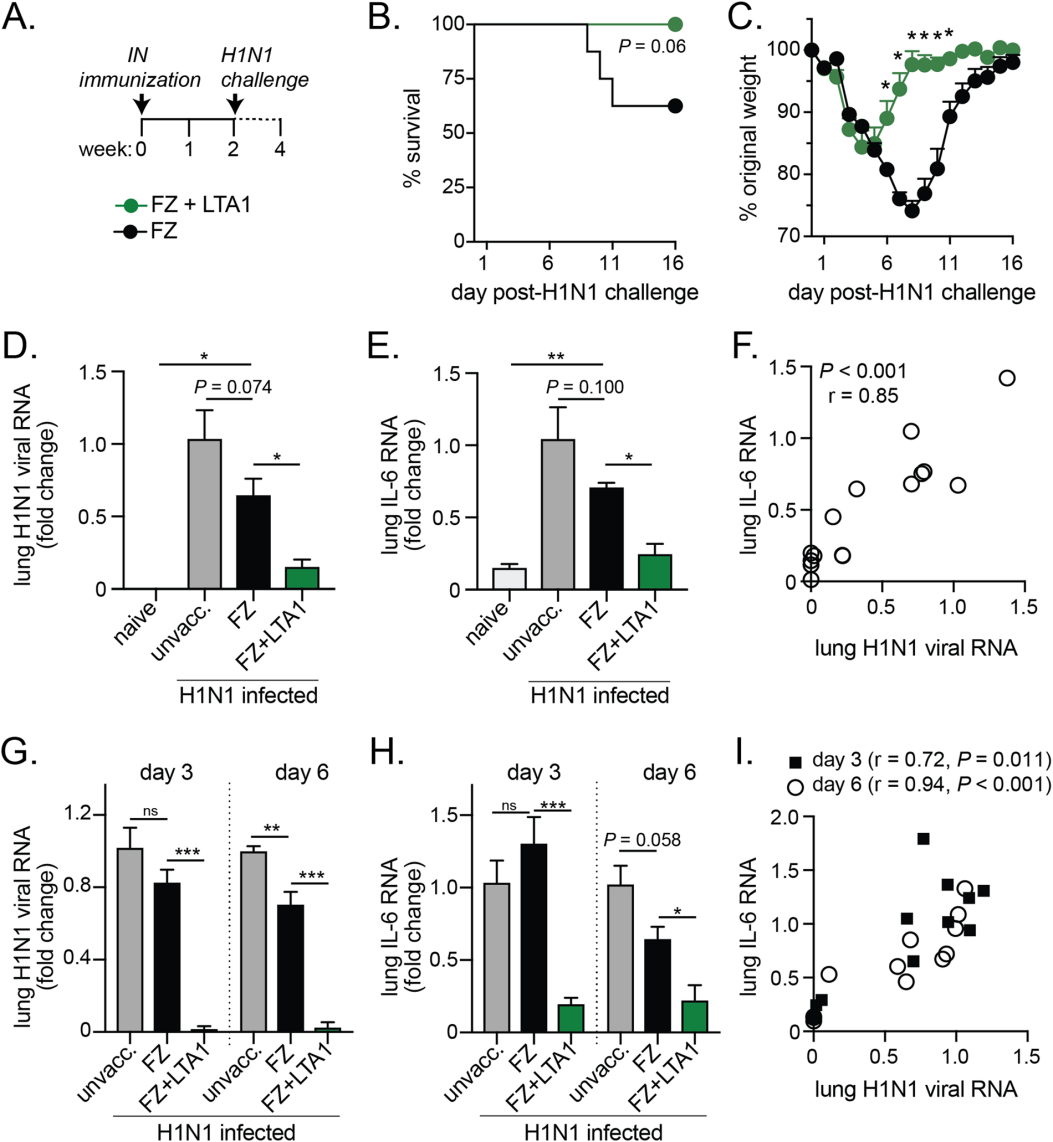
LTA1 adjuvant enhances protection from H1N1 flu challenge after a single or prime/boost vaccination. (**A**) Schematic of mice prime vaccination and challenge: groups of C57Bl/6 mice (7–9 weeks of age) were left unvaccinated (unvacc.) or IN immunized with 3.6 μg HA-content FZ alone (FZ) or with 10 μg LTA1 (FZ + LTA1). After 14 days, mice were challenged with 2xLD50 H1N1. (**B**) Survival of H1N1 challenge and (**C**) weight change (as % from day of H1N1 challenge) in vaccinated young adult mice (n = 8). (**D**) Lung H1N1 viral RNA and (**E**) lung IL-6 RNA levels by qRT-PCR and quantified as fold change to unvaccinated/H1N1 infected mice with naïve mice added as an additional control were analyzed from day 6 post-H1N1 challenged mice (n = 3–4). (**F**) Correlation of lung RNA data from D&E with Spearman correlation coefficient (r) and P-value indicated. (**G**) Lung H1N1 viral RNA and (**E**) lung IL-6 RNA levels by qRT-PCR and quantified as fold change to unvaccinated/H1N1 infected mice were analyzed from day 3 or day 6 post-H1N1 challenged mice (n-4) previously vaccinated prime/boost (day 0, 21) and challenged on day 35. (**I**) Correlation of lung RNA data from G&H with Spearman correlation coefficient (r) and *P*-value indicated. Data reported as mean + SEM with significance indicated by log-rank (survival), t-test with Bonferroni-Dunn correction (weight), or by one-way ANOVA with Dunnett’s post-test compared to FZ group (*P ≤ 0.05, **P ≤ 0.01, ***P ≤ 0.001, ns = not significant).

We next examined if mice immunized with FZ and LTA1 adjuvant would improve protection from H1N1 challenge. First, we immunized mice with IN delivery of FZ alone or with 10 μg LTA1 (FZ + LTA1), then challenged them 2 weeks later with lethal pandemic Influenza strain A/California H1N1 (Fig. 4A). All mice were monitored for mortality and morbidity (e.g. weight loss). We found this single FZ + LTA1 immunization resulted in 100% protection from H1N1 mortality compared with only ~60% survival after FZ immunization (*P* = 0.06, Fig. 4B). FZ + LTA1 animals initially exhibited weight loss on days 2–4 post-challenge but recovered more rapidly than did FZ-immunized animals (*P ≤ 0.05 for weight loss on study days 6–11 post-challenge, Fig. 4C). We also observed improved protection from H1N1 survival and weight loss in a prime/boost model with 24-month old mice immunized with FZ + LTA1 compared to FZ (Supplementary Fig. 4). In conclusion, LTA1 adjuvant improves vaccine-mediated protection from H1N1 challenge in mice.

### LTA1-mediated protection from H1N1 flu challenge is associated with lower lung viral titers, acute-phase response IL-6 cytokine, and higher serum IgM, IgG, and IgA antibodies

We next wanted to confirm our survival and weight loss results by evaluating if viremia or acute-phase response cytokine IL-6 during H1N1 challenge would be similarly improved by the addition of LTA1 adjuvant to vaccination. To do so we tested level of H1N1 viral RNA or IL-6 RNA in the lungs of mice day 3 or day 6 post-challenge comparing unvaccinated, FZ-vaccinated, or FZ + LTA1 vaccinated groups after 1 or 2 immunizations (Fig. 4D–I). In all instances, we observed significantly lower levels of lung H1N1 viral RNA in the FZ + LTA1 mice compared the FZ only group (*P* < 0.05 for day 6 post-H1N1 in primed animals; *P* < 0.001 for day 3 or 6 post-H1N1 in prime/boosted animals; Fig. 4D,G). Similarly, we observed significantly lower levels of lung IL-6 RNA in the FZ + LTA1 mice compared the FZ only group (*P* < 0.05 for day 6 post-H1N1 in primed animals; *P* < 0.001 for day 3 or *P* < 0.05 day 6 post-H1N1 in prime/boosted animals; Fig. 4E,H). Using data collected from all animals, we also observed that the level of viral RNA significantly correlated to lung IL-6 for each experimental conditions (Spearman’s *P* < 0.001 and r > 0.7; Fig. 4F,I).

**Figure 5:**
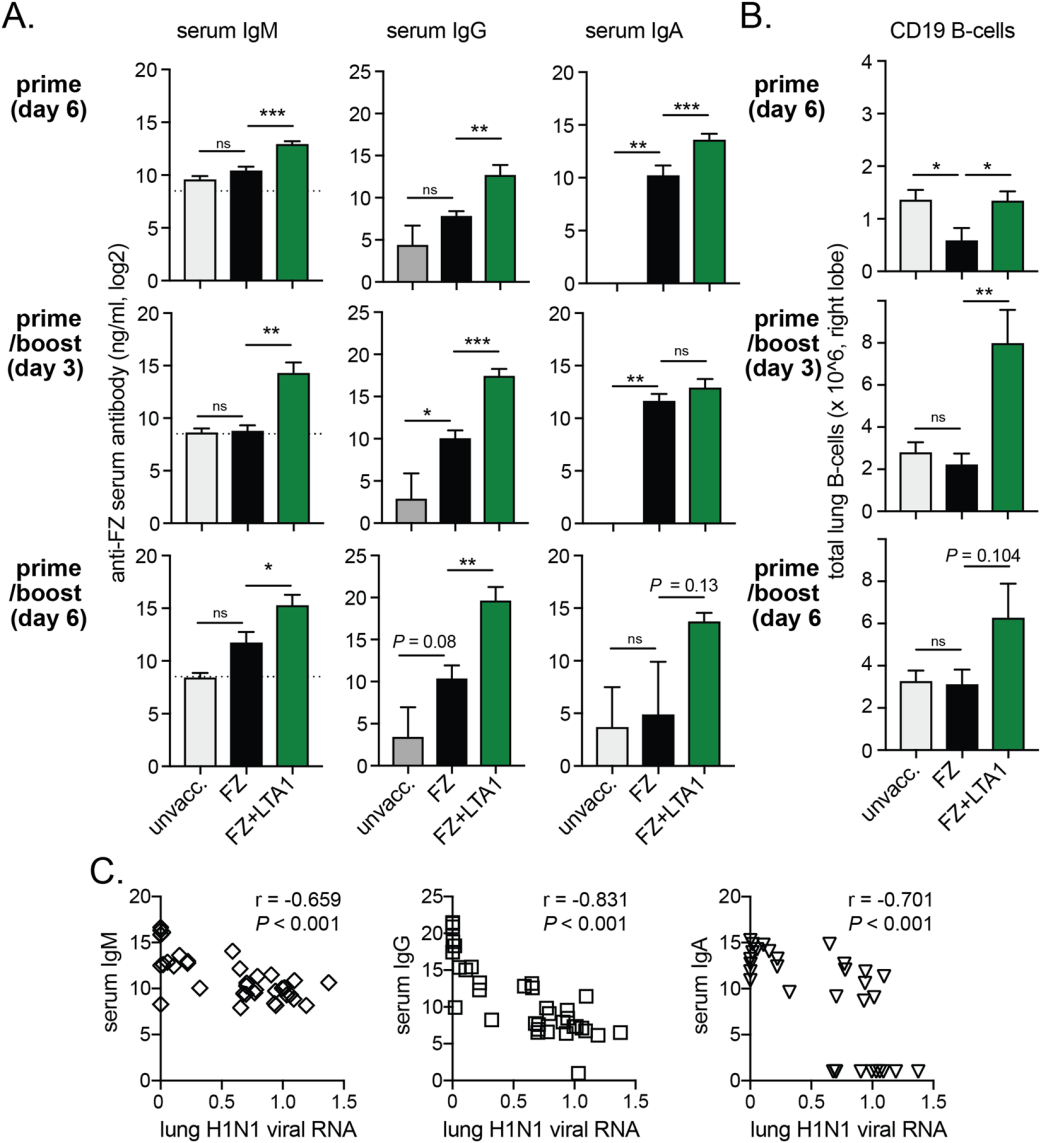
LTA1-mediated protection from H1N1 flu challenge is associated with humoral immunity post-challenge (serum IgM, IgG, and IgA antibodies, lung CD19 B-cells). Groups of C57Bl/6 mice (7–9 weeks of age, n = 4) were left unvaccinated (unvacc.) or IN immunized with 3.6 μg HA-content FZ alone (FZ) or with 10 μg LTA1 (FZ + LTA1) on day 0 (prime) or days 0 and 21 (prime/boost). All mice were challenged 14 days after the last vaccination with 2xLD50 H1N1. (**A**) Serum anti-FZ IgM, IgG, or IgA levels analyzed from day 3 or day 6 post-H1N1 challenged mice by ELISA (dotted line on IgM graphs at level of non-specific/background binding). (**B**) Total lung B-cells from day 3 or day 6 post-H1N1 challenged mice quantified by cytometric analysis of homogenized right lobe lung cells gated live-cell positive, dump channel (Gr1, CD11c) negative and CD19+. (**C**) Correlation of all post-challenge serum IgM, IgG, or IgA data to lung H1N1 viral RNA levels with Spearman correlation coefficient (r) and *P*-value indicated. Bars at mean + SEM with significance by one-way ANOVA with Dunnett’s post-test compared to FZ group (*P ≤ 0.05, **P ≤ 0.01, ***P ≤ 0.001, ns = not significant).

We also evaluated serum FZ-specific IgM, IgG, and IgA antibody responses and number of total CD19+ B-cells in the lungs from these animals post-challenge (Fig. 5A,B). We observed significant changes in post-challenge levels of serum IgM, IgG, and lung B-cells post-challenge in mice immunized with FZ + LTA compared to those immunized just with FZ after 1 immunization or 2 immunizations (*P* < 0.05 or greater). Serum IgA responses were also elevated in the FZ + LTA1 group, but only significantly different from FZ at 6-days post-H1N1 challenge after prime immunization (*P* < 0.05). Using data collected from all animals all timepoints, we also observed that the level of serum IgM, IgG, or IgA significantly and inversely correlated to levels of lung H1N1 viral RNA (Spearman’s *P* < 0.001 and r < −0.65; Fig. 5C). Thus, LTA1 immunized animals were better protected from high levels of H1N1 viral replication as well as viral associated acute phase responses IL-6 cytokine secretion while exhibiting higher levels of humoral immune responses post-challenge (anti-FZ serum antibodies).

### LTA1-mediated protection from H1N1 flu challenge does not require B-cells

**Figure 6:**
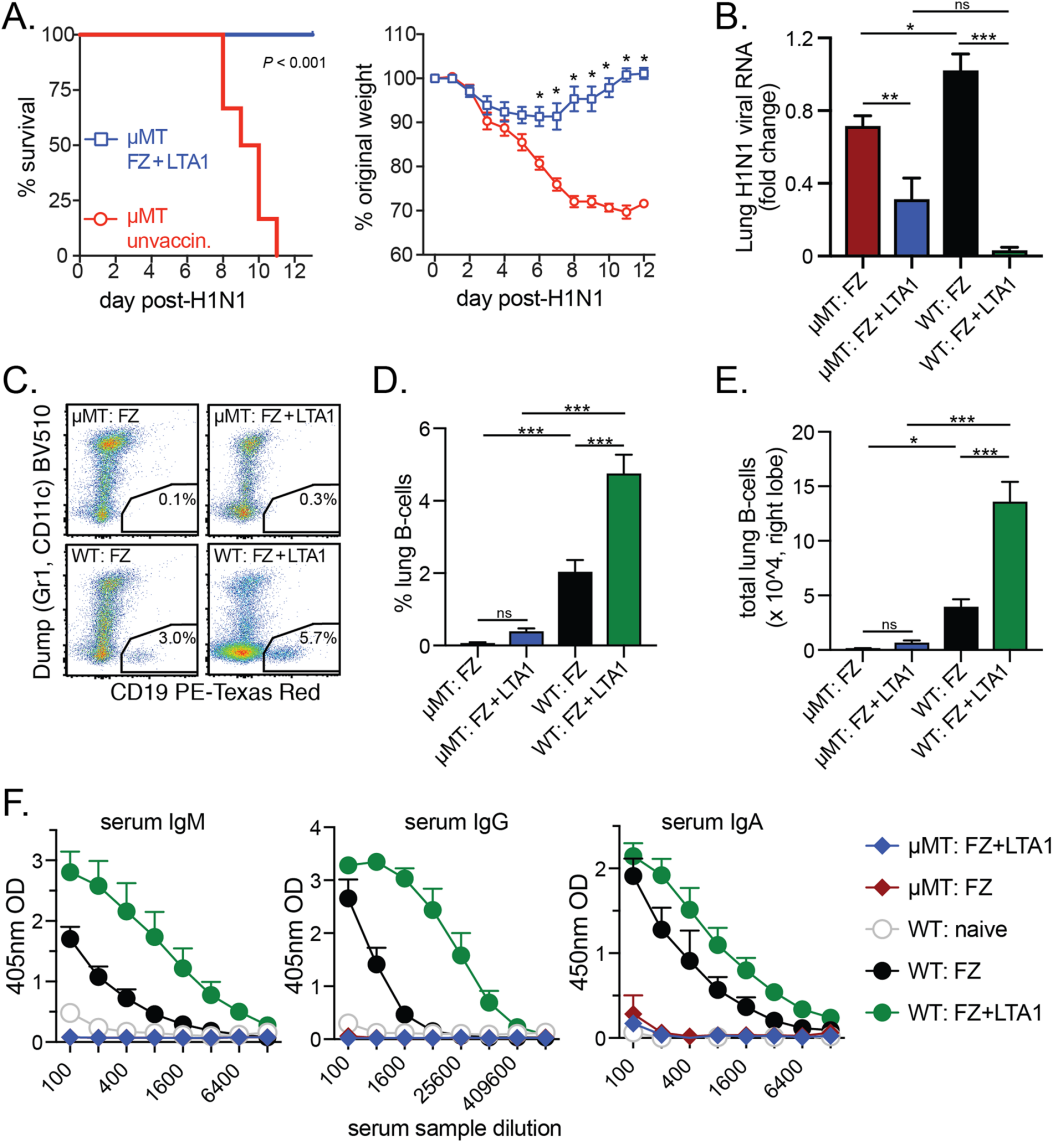
B-cells are not required for LTA1-mediated vaccine protection from H1N1 flu challenge. Groups (n = 6–7) of μMT mice or C57Bl/6 (WT) mice were left unvaccinated (unvaccin.) or IN immunized twice with 3.6 μg HA-content FZ alone (FZ) or with 10 μg LTA1 (FZ + LTA1) then challenged with 2xLD50 H1N1. (**A**) Survival of H1N1 challenge (left graph) and weight change (as % from day of H1N1 challenge, right graph) in FZ + LTA1 or unvaccin. μMT mice. (**B**) Lung H1N1 viral RNA analyzed from day 6 post-H1N1 challenged μMT and WT mice in indicated groups by qRT-PCR and quantified as fold change to WT: FZ group. (**C**) Representative dot plots of live-gated lung cells for dump channel (Gr1, CD11c) versus CD19 with B-cell gates from 6 days post-H1N1 challenge μMT and WT mice in indicated groups. (**D**) Compiled % lung B-cells from 6 days post-H1N1 challenge μMT and WT mice in indicated groups. (**E**) Compiled total lung B-cells (right lobe) from all cells collected 6 days post-H1N1 challenge from μMT and WT mice in indicated groups. (**F**) Serum anti-FZ IgM, IgG, and IgA ELISA raw data from day 6 post-H1N1 challenged μMT and WT mice in indicated groups (naïve = uninfected/unvaccinated mice). Data reported as mean + SEM with significance indicated by log-rank (survival), t-test with Bonferroni-Dunn correction (weight), or by one-way ANOVA with Bonferonni’s post-test for selected groups as shown (*P ≤ 0.05, **P ≤ 0.01, ***P ≤ 0.001).

B-cells are critical to protection from influenza, as mice lacking mature B-cells (μMT mice) have worse survival outcomes post-challenge^31^. In addition, changes in serological responses (e.g., HA-neutralizing antibody titers) are the primary correlate of vaccine efficacy for existing or new clinical influenza vaccines^32^. We also observed higher levels of B-cells and serum antibodies in FZ + LTA1 vaccinated animals than in other tested groups (Fig. 5). To investigate if the protective effects of LTA1 adjuvant flu vaccine require intact humoral responses, we immunized μMT mice with FZ + LTA1 twice on days 0, 21 and then compared their responses to lethal H1N1 infection on day 35 with that of naïve μMT mice. Immunized mice were completely protected from mortality after flu challenge (*P* < 0.001) and also exhibited minimal weight loss compared with naïve μMT mice (significant beginning day 6 post-infection; Fig. 6A).

To confirm these results, we next evaluated animals from μMT or C57Bl/6 (wild-type or WT) mice 6 days after H1N1-challenge who had been immunized prime/boost with either FZ or FZ + LTA1. Similar to previous results (Fig. 4), we observed lower lung H1N1 viral levels in FZ + LTA1 mice compared with FZ in either μMT or WT strains (*P* < 0.01 in μMT mice or *P* < 0.001 in WT mice, Fig. 6B). In addition, all μMT mice irrespective of immunization history had significantly reduced levels of % or total lung CD19+ B-cells (Fig. 6C–E) and no detectable anti-FZ serum IgM, IgG, or IgA 6-days post-H1N1 challenge (Fig. 6F). No differences in B-cells or antibody levels were observed between FZ or FZ + LTA1 groups in μMT mice. Thus, despite previously observed humoral effects with FZ + LTA1 immunization, the protective effects of LTA1-adjuvanted flu vaccine is not dependent upon B-cells.

### LTA1-mediated protection from H1N1 flu challenge is associated with lung CD4 T-cells

**Figure 7:**
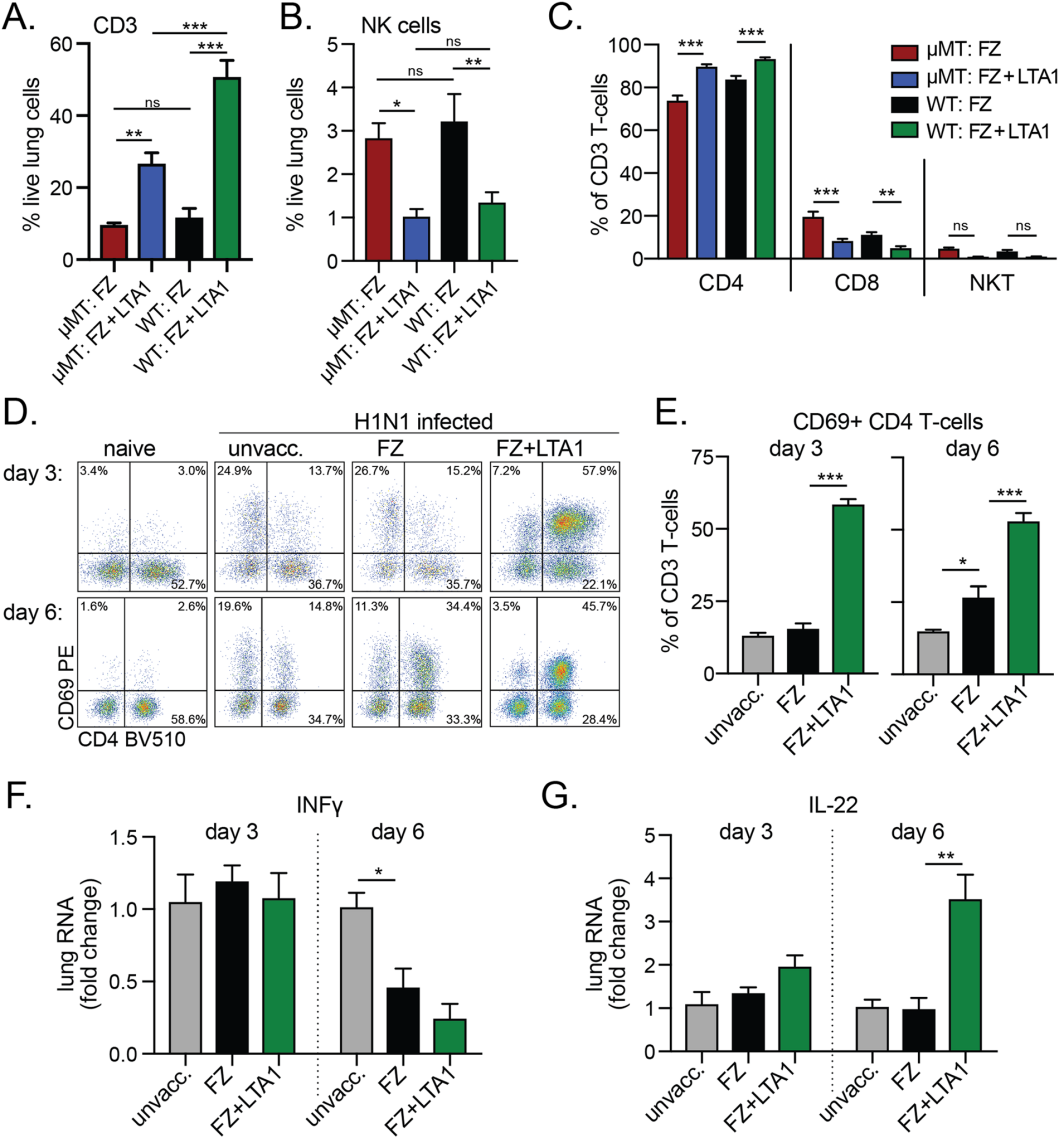
Activated CD4 T-cells are found in higher levels in LTA1 vaccinated animals post-H1N1 challenge. Groups (n = 6–7) of μMT mice or C57Bl/6 (WT) mice were left unvaccinated or IN immunized twice with FZ or FZ + LTA1, then all mice challenged with 2xLD50 H1N1. (**A**) Compiled % CD3 T-cells of live-gated lung cells (CD11c−, CD3+) 6 days post-H1N1 challenge from μMT and WT mice in indicated groups. (**B**) Compiled % NK cells of live-gated lung cells (NK1.1+, CD11c−, CD3−) 6 days post-H1N1 challenge from μMT and WT mice in indicated groups. (**C**) Compiled % CD4 (CD8−, NK1.1−), CD8 (CD8+, NK1.1), or NKT (CD8−, NK1.1+) cells of CD3 lung T-cells 6 days post-H1N1 challenge from μMT and WT mice in indicated groups. (**D**) Representative dot plots of CD4 vs CD69 markers expression on CD3 lung T-cells from indicated groups of WT mice 3 or 6 days post-H1N1 challenge. (**E**) Compiled % CD4+ CD69+ of CD3 T-cells (CD8−, NK1.1−, CD4+) from indicated groups of WT mice 3 or 6 days post-H1N1 challenge. (**F**) Lung INFγ viral RNA levels analyzed from day 6 post-H1N1 challenged WT mice (n = 3–4) by qRT-PCR and quantified as fold change to unvaccinated/H1N1 infected mice. (**G**) Lung IL-22 viral RNA levels analyzed from day 6 post-H1N1 challenged WT mice (n = 3–4) by qRT-PCR and quantified as fold change to unvaccinated/H1N1 infected mice. Bars at mean + SEM with significance by one-way ANOVA with Bonferonni’s post-test for selected groups as shown (**A**–**C**) or Dunnett’s post-test compared to FZ group (E-G, *P ≤ 0.05, **P ≤ 0.01, ***P ≤ 0.001, ns = not significant).

Since B-cells were not required for LTA1 mediated protection from flu challenge, we next evaluated changes to other immune cell populations in the lungs of μMT or WT mice 6 days after H1N1-challenge who had been immunized prime/boost with either FZ or FZ + LTA1. We observed higher levels of % CD3 T-cells and lower % of levels of NK cells recruited into the lungs of FZ + LTA1 μMT or WT groups post-challenge (*P* < 0.05 or greater, Fig. 7A,B). Within this CD3 T-cell population in both mouse strains, the proportion of CD4 T-cells was greater in FZ + LTA1 groups (*P* < 0.001, Fig. 7C), while % of CD8 T-cells or NKT cells were smaller or not-significantly different. Further evaluation in WT mice, indicated that CD4 T-cells in the FZ + LTA1 animals were also expressing significantly high levels of early activation marker CD69 than unvaccinated or FZ mice on days 3 and 6 post-H1N1 challenge (*P* < 0.001, Fig. 7D,E). Evaluation of INFγ RNA levels in the lungs of these mice was not significantly different between vaccinated mice groups (Fig. 7F), indicating no evidence for stronger Th1 responses. In contrast, slightly higher levels of IL-22 RNA were observed in FZ + LTA1 mice compared with FZ (*P* < 0.01 for day 6, Fig. 7G). IL-22 secretion by T-cells has previously been shown to be critical for epithelial repair during influenza infection^33,34^, but has not been explored as a mechanism in enhanced protection from enterotoxin based adjuvants. Thus, the protective effects of LTA1-adjuvanted flu vaccine are associated with CD4 T-cell activation and possibly IL-22 secretion.

## Discussion

In this study, we identified LTA1 as a safe, effective adjuvant for IN delivery with the potential to overcome past clinical safety issues observed with the LT-adjuvant flu vaccine. This is the first report of LTA1 enhancing vaccine protection against flu challenge or any infectious challenge. This clearly refutes the long-held belief that the A1 domain of LT is not stable enough to bind to or be taken up at mucosal surfaces and mediate immunologic stimulation. Additionally, this is the first study to demonstrate safety of an LT-based IN adjuvant using behavioral testing as evidence for lack of neurological toxicity in comparison with holotoxin. Lastly, we provide insight into the protective mechanisms of LTA1, identifying that B-cells are not required for adjuvanted vaccine protection against flu challenge but CD4 T-cells are highly activated. These results have direct implications for vaccine development and use of LTA1 as a novel IN adjuvant.

Enterotoxin-based adjuvants are well known inducers of mucosal immunity, but their use in IN vaccination was hindered by increased risk of Bell’s palsy (estimated at 13 cases per 10,000 vaccine recipients) compared with non-vaccinated individuals^14^. One benefit of this past clinical observation is that other severe adverse events (e.g., local site reactogenicity, increased risk for cancer, birth defects, or pulmonary complications etc.) were never observed in pre-clinical and clinical studies^14,26^. Thus, the specific safety risks that must be mitigated for a safe LT-based IN adjuvant are clearer than with an untried adjuvant. There is no pre-clinical model of Bell’s palsy or other assessment of cranial nerve 1 reported with enterotoxin use. Therefore, we utilized the olfactory habituation-dishabituation test that assesses neurotoxicity through changes to cranial nerve 7 and olfactory system function (Fig. 1)^23^. While we previously speculated that removal of the B-subunit would limit neurological toxicity^24^, this is the first demonstration of nontoxicity after nasal delivery of a LT-based adjuvant (at a 3–30x expected dose) using this methodology. Combined with our previous data demonstrating poor antigenic potential^25^, this indicates LTA1 is an attractive option to overcome past clinical trial safety concerns. It is also worth mentioning, that LTA1 is also distinct from any other proposed IN enterotoxin adjuvant because it is based on LT protein and has no specific binding or cell-targeting component. For example, CTA1-DD has also been presented as an IN adjuvant but it is composed of a B-cell targeting synthetic component based on protein A and the A1 domain of CT (which is differs from LT by level of enzymatic activity and quality of immune responses)^35,36^. However, continued investigations with LTA1 both for neurological safety and in comparison with related adjuvants are warranted.

In our experiments, we utilized a flu vaccination model to determine the ability of LTA1 to improve vaccine efficacy. However, our results in this model are relevant since optimal protection against influenza has yet to be achieved^6^. Seasonal and pandemic influenza (flu) are considered major public health threats despite annual vaccine campaigns^37,38^. Addition of an adjuvant can overcome limited vaccine efficacy in high-risk populations (i.e., young, elderly) and also allow dose-sparing in case of vaccine shortages due to the 6–9 month production time for new vaccines^37,39,40^. Lastly, while traditionally IN vaccines may not perform as well as injectables, they often are a popular needle-free option, as observed with FluMist® vaccination campaigns in US schools^41^. Thus, an adjuvanted IN vaccine may improve vaccine efficacy particularly for high-risk groups and improve logistics of mass delivery during seasonal disease or pandemic outbreaks. In this study, we were able to show IN vaccination with an optimized dose of LTA1 admixed with an inactivated virus antigen improved survival, weight loss, and viremia from lethal H1N1 challenge (Fig. 4, Supplementary Fig. 4). Thus, LTA1, like its parent molecule LT, can boost immunity to flu antigens and has potential as a novel adjuvant for IN vaccine strategies.

We observed increases in humoral immunity in LTA1 adjuvanted vaccination that correlated with reduced lung viremia (Fig. 5). Despite this observation, μMT mice immunized twice with LTA1 + FZ were protected from severe weight loss and death from lethal H1N1 challenge and still exhibited reduced lung viremia (Fig. 6). Immunized mice did exhibit a slight decrease in weight post-challenge (5–10%) that differed from that observed in non-transgenic adult mice (seen in Supplementary Fig. 4). μMT mice have a disruption in the IgM heavy chain and therefore don’t develop mature B-cells and are estimated to have at least 50-fold more sensitivity to influenza infection^31,42^. This indicates that classic B-cell functions, including antigen presentation and neutralizing antibodies are not required for LTA1 adjuvant effects, though B-cell responses may be necessary for maximal adjuvant-mediated protection. In both μMT and WT mice, we observed high levels of CD4 T-cells in the lungs during infection (Fig. 7). This suggests that LTA1-mediated protective responses may be dependent upon CD4 T-cells or both B-cells and CD4 T-cells may play redundant roles in protection that should be explored in future studies.

In conclusion, these results support LTA1 as a unique mucosal adjuvant that has broad potential for flu and other vaccines. The impetus for this adjuvant development comes as successful control of infectious diseases through vaccination and other public health measures has reduced levels of circulating infections, thereby widening the gap between acceptable risks of side effects versus benefits from disease protection afforded by vaccination. This ever-evolving dialog alters how vaccines are formulated and scheduled, even precluding the use of older, effective live-attenuated vaccines or resulting in the phasing out of whole-killed vaccines due to reactogenicity and/or risk of disease, such as with pertussis and polio vaccines^2–5^. However with altered vaccine formulations, tradeoffs in immunity are being created, including gaps in protection observed currently with both polio and pertussis vaccines that protect against clinical disease but not transmission. Adjuvants such as LTA1 that allow needle-free delivery and can promote mucosal immunity to subunit vaccination^24^ or inactivated vaccines (as reported here) can play the critical role of filling these key gaps in protection in the continuously changing landscape of vaccination and disease prevention policies.

## Methods

### Vaccines and adjuvant proteins

LTA1 and CT were produced from *E*. *coli* clones expressing recombinant protein as previously described^25,43^. Proteins were stored lyophilized and freshly resuspended prior to use (CT) or kept frozen at −20 °C until use (LTA1). The endotoxin content of all proteins was < 1 EU/mg. The commercial trivalent flu vaccine: 2015–2016 Fluzone High-Dose® (FZ, Sanofi Pasteur, Swiftwater, PA) was purchased in 2015 from the Tulane University Hospital Pharmacy and contained 180 μg/0.5 ml hemagglutinin (HA) from equal parts of A/California/07/2009 H1N1, A/Switzerland H3N2, and B/Phuket strains.

### Mice

Seven-to-9-week old C57BL/6 female mice were purchased from The Jackson Laboratory (Bar Harbor, ME). For aged mice experiments, Balb/c mice were purchased at 6-to-8-weeks of age from NCI Charles River and aged in-house. Male and female μMT mice (strain B6.129S2-Ighmtm1Cgn/J) were originally purchased from The Jackson Laboratory and bred in house for experiments. All mice were housed in sterile set-up with filter-top cages. Rodents were fed normal chow, with addition of weekly supplemental wet diet gel 76 A (ClearH2O, Portland, ME) for aged mice experiments. Mice were euthanized by CO_2_ inhalation and exsanguination (immunization studies) or ketamine/xylazine overdose (flu and olfactory studies). All mouse experiments were approved by the Tulane University Institutional Animal Care and Use Committee and performed in accordance with relevant guidelines and regulations.

### Olfactory habituation-dishabituation test & nasal cavity histology

Mice were individually housed and IN treated or left naive and tested by olfactory habituation-dishabituation 24 h later as previously described^23^. Treatments included 30 μg LTA1 (0.5 mg/ml) or 1–30 μg CT (1 mg/ml). Trials were performed with 2cmx2cm filter paper freshly infused with 20 μl mineral oil (Millipore Sigma), cheese trout bait (Berkley, Columbia, SC, diluted 1:2 in ethanol), or shrimp oil (Atlas Mike’s, Fort Atkinson, WI) and by recording times investigating paper within ≤1 mm. After testing, select mice (n = 3/group) were euthanized using ketamine/xylazine and nasal cavities dissected according to established guidelines^27^ for formalin fixation, paraffin embedding, sectioning, H&E staining, and blindly analyzed by a pathologist using a score added for all three nasal sections per mouse (0 = normal, 1 = mild neutrophil inflammation and/or edematous changes, 2 = moderate degenerative or proliferative lesion, 3 = severe lesions with extensive damage to tissue architecture). Mast cells were analyzed using standard toluidine blue staining of paraffin embedded sections, counting total number purple cells per 100x microscopic field area added for two nasal sections (0 = <5 cells per area, 1 = 5–15/area, 2 = 15–25/area; 3 = 25–35/area; 4 = >35/area).

### Animal vaccination & H1N1 challenge

Groups of mice (n = 4–8) were immunized with 3.6 μg FZ (or 1.2 μg of H1N1) containing 0–10 μg LTA1 diluted in PBS (20–30 μl total volume) IN by pipetting directly into nares of mice anesthetized with ketamine/xylazine or (aged mice only) inhaled isoflurane. Two weeks after the last immunization, mice were euthanized for sample collection or anesthetized again and challenged IN with mouse-adapted influenza A/California/07/2009 (H1N1) diluted in PBS (50 μl total volume) at median lethal (1xLD50) or lethal (2xLD50) doses optimized for Balb/c or C57Bl/6 mouse strains. Mice were monitored for weight daily with loss of 30% initial body weight the endpoint for euthanasia. Some surviving mice were euthanized and perfused by cardiac puncture with saline, prior to inflation of lungs with 10% formalin for paraffin embedding, sectioning and H&E staining.

### Mouse sample collection & processing

Serum was collected by cardiac puncture. BAL samples were collected by tracheal catherization and lavage with 1 ml PBS + protease inhibitor cocktail (Roche, Mannheim, Germany). Spleens were placed into C-tubes and homogenized via gentleMACS dissociator (Miltenyi Biotec, Bergisch Gladbach, Germany). After filtration and red blood cell lysis (ACK lysis buffer, Millipore Sigma, St. Louis, MO), 1 × 10^6^ cells were plated in 200 μl RPMI-10% FBS for antigen restimulation then supernatants harvested after 72 h culture for cytokine analyses.

### Antibody ELISAs & cytokine measurements

Anti-FZ IgG1, IgG2a, and IgA antibody ELISAs were performed using CoStar 96-well flat bottom plates (Washington, DC) similar to as previously described^13,24^. Briefly, wells were coated with 1 µg/ml FZ or recombinant standards (IgG1, IgG2a (Millipore Sigma) or IgA (Southern Biotech, Birmingham, AL)), incubated with sample dilutions, and detected with AKP-conjugated anti-mouse IgG1, IgG2a (BD Biosciences, Franklin Lakes, NJ) or HRP-conjugated anti-mouse IgA (Millipore Sigma). Results reported as ELISA units/ml (EU/ml) are averaged interpolated values around the standard curve midpoint. Cytokines were analyzed with thawed supernatants using Mouse CD8 T-cell Panel (Millipore Sigma) with a Bioplex 200 array reader (Bio-Rad, Hercules, CA).

### Lung RNA analyses

Lungs were harvested at 3- or 6-days after infection. Left lung tissue from infected and control mice was minced and frozen at 10 μl/mg in RLT buffer (Qiagen, Valencia CA). 20 mg lung tissue were homogenized with Lysing Matrix D-tubes using Fastprep24 using two 15 sec cycles and RNA extracted following Qiagen RNeasy Plus Mini Kit instructions. cDNA was generated using iScript reagents and protocol (Bio-Rad) and analyzed by real-time PCR using the Bio-Rad CFX connect detection system. H1N1 virus levels were quantified using Influenza A primer and probe set obtained through BEI Resources, NIAID, NIH: Influenza Virus Real-Time RT-PCR Assay, NR-15592. Cytokines were quantified using PrimePCR^TM^ Probe Assays for mouse Ifng, Il6, and Il22 (Bio-Rad). GAPDH was used as a housekeeping gene and quantified using Mm.PT.39a.1 qPCR assay (IDT, Coralville, IA). All data was quantified as fold-change mRNA by ΔΔCt method relative to control mice. Any outliers identified by Grubbs analysis (maximum 1 per dataset) were removed from final analyses. For a full primer list see Supplementary Table 1.

### Lung cytometric analyses

Right lung tissue was harvested, placed in RPMI-1640, minced with scissors and digested for 1 h at 37 °C with 100 rpm shaking in 4 ml of digestion solution containing 2 mg/ml Type IV Collagenase (Sigma) and 80U/ml DNAse I (Sigma). Digested lungs were passed through a 70 µm filter, RBC lysed (Quality biological Gaithersburg, MD), and washed in PBS with 1% BSA and 2 mM EDTA. For immunofluorescence staining, single cell lung suspensions were stained using fixable viability dye eFluor 780 or eFluor 450 (ThermoFisher Sci., Waltham, MA) and antibodies to surface markers including: anti-mouse CD19 conjugated to PE-Texas Red (clone 6D5) CD11c PerCP-Cy5.5 (N418) (ThermoFisher Sci.), CD11c BV510 (N418), Ly-6G/Ly-6C BV510 (RB6-8C5), NK-1.1 BV605 (PK136), CD4 BV510 (RM4-5), CD3 AF700 (17 A2), CD8 APC/Fire 750 (53-6.7) (BioLegend, San Diego, CA); and CD69 PE (H1.2F3, BD Biosciences, San Jose, CA). Stained cells were fixed with 2% paraformaldehyde (PolySciences Inc., Warrington, PA). Cytometric analyses were performed with a BD LSRFortessa and analyzed using FlowJo v10 software (Ashland, OR).

### Statistical analysis

Statistical analysis was performed using GraphPad Prism v.7.0 or 8.0 (La Jolla, CA). Antibody and cytokine data were transformed for normalcy and homogeneity of variance prior to analysis (normalcy tested by Sharpio-Wilk test). Statistics were performed using t-test using Bonferroni-Dunn correction or one- or two-way ANOVA with two-tailed Dunnett’s or Bonferroni post-tests for all compared to control or selected pairs respectively. Survival analyses were performed using log-rank test. Correlations were performed with Spearman’s rank-order correlation. Final figures were prepared using Adobe Illustrator software (San Jose, CA).

## Supplementary information

Supplementary video

Supplementary Info

## Supplementary information

is avialable for this paper at 10.1038/s41598-019-51356-w.
