# Supplementary material for: LTA1 is a safe, intranasal enterotoxin-based adjuvant that improves vaccine protection against influenza in young, old and B-cell-depleted (μMT) mice
Source: Sci Rep. 2019 Oct 22;9:15128. doi: 10.1038/s41598-019-51356-w (PMC6805908; doi:10.1038/s41598-019-51356-w)
Supplement: Supplementary file 2 — Supplementary Info [file 41598_2019_51356_MOESM2_ESM.doc]

**Supplementary Material**

**LTA1 is a safe, intranasal enterotoxin-based adjuvant that improves vaccine protection against influenza infection in young, old and B-cell depleted (μMT) mice.**

**Authors: E. Valli^1^, A. J. Harriett^1^, M. K. Nowakowska^1^, R. L. Baudier^1^, W.B. Provosty^1^, L. B. Lawson^1^, Y. Nakanishi^2^ and E. B. Norton^1^***

**Supplementary Video 1**. [see separate mp4 file] Compilation of 8x speed video recordings of select trials of the habituation-dishabituation test for mice representative of naïve (untreated), 30µg LTA1 IN or 5-10µg CT IN treatment groups.

**Supplementary Figure 1. Altered olfactory system responses are detected by dose-dependent administration of CT 24h prior to testing.** Individually housed mice were untreated (untx) or anesthetized with ketamine/xylazine and treated IN by pipetting 1-30 µg CT in the nares. After 24h, the habituation-dishabituation test was performed using 3min trials with exposure to filter paper laced with mineral oil, cheese odor or shrimp odor with 15 mn rests in between trials. Individual mouse results are shown for 1, 5, or 30 µg CT with 4-5 mice used for 10 µg CT and untx respectively (mean+SEM) for number of times investigating filter paper during each trial.

**
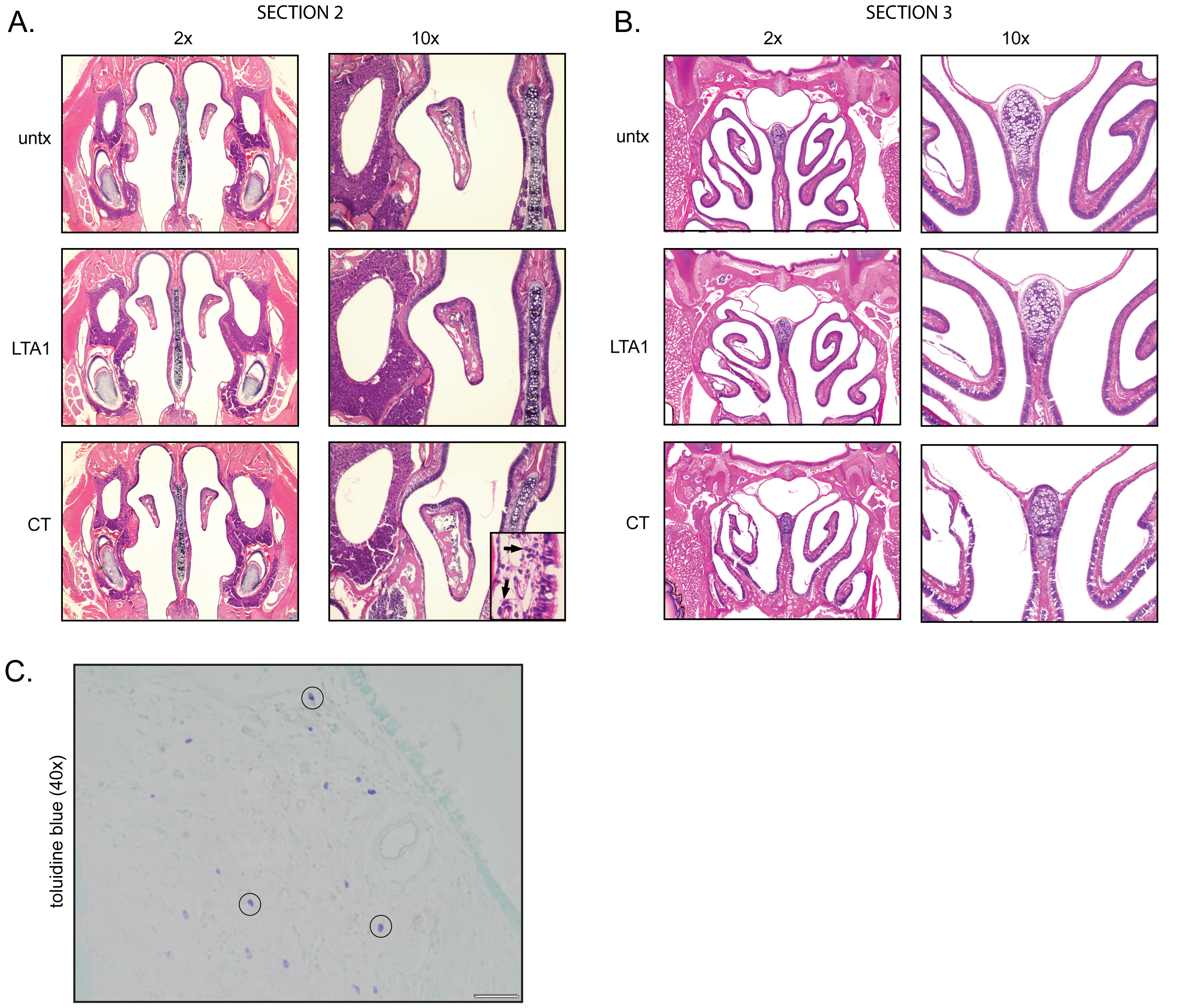
**

**Supplementary Figure** **2**. Representative H&E-stained images of the nasal cavity of mice treated as indicated from section 2 (A, by the incisive papilla) and section 3 (B, by the second molar tooth). Inset arrows indicate neutrophils and mild, acute inflammation occasionally observed in CT-treated mice in section 2 beneath the epithelium within the nasal mucosa. (C) Example toluidine blue staining of nasal tissue with a few mast cells circled.


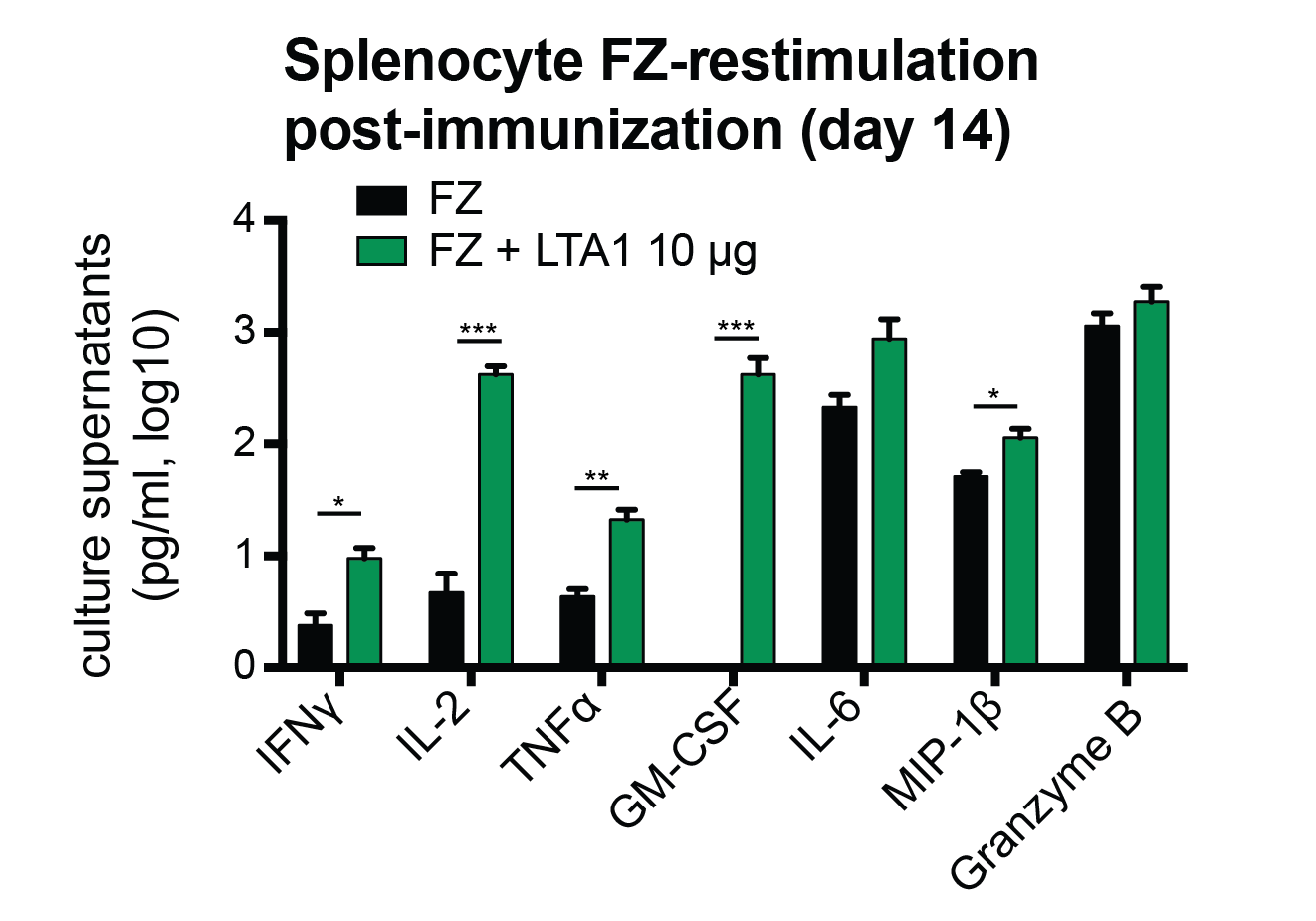


**Supplementary Figure** **3. Immune responses to IN flu immunization are enhanced with LTA1 adjuvant.** Groups of C57Bl/6 mice (n=5) were immunized intranasally with 36 μg HA-content FZ alone or with 10 μg LTA1. Cytokines/proteases (GrzB = granzyme B) from 72h culture supernatants of FZ stimulated splenocytes from mice post-prime (day 14). Significance is indicated by **P* ≤ 0.05, ***P* ≤ 0.01, or ****P* ≤ 0.001 for all groups compared to FZ by t-test with Bonferroni-Dunn post-test.

**Supplemental Figure 4.** **LTA1 adjuvant enhances protection from H1N1 flu challenge in adult and old FZ-vaccinated mice.** (A) Schematic of old mice vaccination and challenge: groups of Balb/c mice, including 7-month adult (n=5) or 24-month old (n=7), were left naïve or IN immunized twice with 3.6 μg HA-content FZ alone or with 10 μg LTA1 (FZ+LTA1) then challenged with 1xLD50 H1N1, a lethal dose in elderly mice. (B) Survival of H1N1 challenge in vaccinated adult and old mice (100% survival in Adult FZ+LTA1 compared with 38% survival in naïve adult, *P* ≤ 0.05; 71% survival in Old FZ+LTA1 compared with 57% in Old FZ and 0% survival in Old naïve mice, *P* = 0.06). (C) Weight change (as % from day of H1N1 challenge, mean+SEM) in vaccinated adult and old mice, with significance for days 5-12 (*) indicated for *P* ≤ 0.05 in FZ+LTA1 group compared to FZ alone by t-test with Bonferroni-Dunn correction (not shown is improved weights on days 4-24 post-challenge between Adult FZ+LTA1 and Adult naïve groups, *P* ≤ 0.05). (D) Weight change of individual mice as shown (with 2/7 in Old FZ+LTA1 group vs 7/7 mice in Old FZ exhibiting severe weight loss). (E) H&E-stained lung sections from representative mice of surviving animals post-challenge. Lymphoid aggregate structures (e.g., iBALT) were observed in LTA1 immunized animals and is indicated with arrows or flu-damaged tissue (*).

| **Table 1. RNA Primers and probes.** | |  |  |  |
| --- | --- | --- | --- | --- |
| Assay name | Unique Assay ID: | |  | Company |
| IL-6 | qMmuCEP0054186 | | PrimePCR | Biorad |
| IL-22 | qMmuCEP0060632 | | PrimePCR | Biorad |
| IFN-g | qMmuCIP0029255 | | PrimePCR | Biorad |
| GAPDH | Mm.PT.39a.1 | | PrimeTime | IDT |
| Influenza A Probe | NR-15593 |  |  | Bei resources |
| Influenza A Forward Primer | NR-15594 |  |  | Bei resources |
| Influenza A Reverse Primer | NR-15595 |  |  | Bei resources |
